# The Human Foot

**Published:** 1889-12

**Authors:** 


*The Human Foot*. By THOMAS S. ELLIS. Pp. 120. London: J. & A. CHURCHILL. 1889.

This work is clearly the offspring of much thought and considerable study; and here and there is original as well. Although of that somewhat dangerous class which appeals to the public as well as to the profession, the book is entirely commendable in tone as well as in matter. Mr. Ellis makes out clear cases of error against sculptors, military men, and bootmakers; and his views, in our opinion, are as correct as they are novel in each instance. The work is eminently practical, having definite aims towards the improvement of the arts of progression and sculpture and shoeing, and should be read by all therein interested. We being, like most men, interested in all these, as well as in surgical anatomy, have perused the work with interest and profit; and we have no doubt that many of our readers will do the same.

